# Intracranial Gliofibroma: A Case Report and Review of the Literature

**DOI:** 10.1155/2014/165025

**Published:** 2014-07-13

**Authors:** Patricia Gargano, Graciela Zuccaro, Fabiana Lubieniecki

**Affiliations:** ^1^Department of Pathology, Garrahan Pediatrics Hospital, Combate de los Pozos 1881, C1245AAM Buenos Aires, Argentina; ^2^Department of Neurosurgery, Garrahan Pediatrics Hospital, Combate de los Pozos 1881, C1245AAM Buenos Aires, Argentina

## Abstract

Gliofibroma is a rare tumor with biphasic morphology, commonly occurring in the first two decades of life. Currently, the tumor is not listed as a distinct entity in the current World Health Organization (WHO) classification of central nervous system tumors. As its biological behavior, histogenesis, and prognostic factors are still debated, the aim of this paper was to describe a case of a gliofibroma and to update the data about these lesions. Hence, we present here clinical symptoms, pathological findings, and evolution observed in a child with gliofibroma. A 10-year-old girl with seizures was referred for study. Neuroimaging showed a hemispheric hyperdense tumor with little peritumoral edema and no mass effect. The tumor was totally removed. Histologically, the tumor consisted of a mixture of glial cells and collagen-rich stroma. Immunohistochemical examination revealed positive staining for GFAP, CD 99, S100, and vimentin. EMA staining showed a paranuclear dot pattern in only few cells in isolated areas. These findings of a glial component with collagenous stroma were consistent with a desmoplastic glioma. Because of the rarity of this entity, we believe it is important to report every case in order to adequately analyze and categorize the tumor in the next WHO classification.

## 1. Introduction

Gliofibroma is a rare bimorphic tumor composed of both glial and mesenchymal components. The entity was first described by Friede in 1978 in a three-year-old child with a tumor located in the medulla oblongata [1]. Since then, only 25 cases have been reported in the pediatric population. The tumor commonly occurs in the young, especially in the first two decades of life. In the 2007 World Health Organization (WHO) classification of tumors of the central nervous system (CNS), this type of pathology was still not listed as a distinct entity. Because of the paucity of available reports on this tumor, its exact nature and behavior are not completely understood. Here, we present a case of hemispheric gliofibroma in a child, describing the clinical features, images observed, follow-up performed, histological findings, and immunohistochemical profile of the pathology and a review of the literature.

## 2. Case Report

A previously healthy 10-year-old girl was referred to the Garrahan Hospital (Buenos Aires, Argentina) because of partial seizures occurring during sleep. She responded well to carbamazepine. On examination, her vital signs were within normal limits. Routine hematology and serum chemistry revealed no abnormalities.

**Figure 1:**
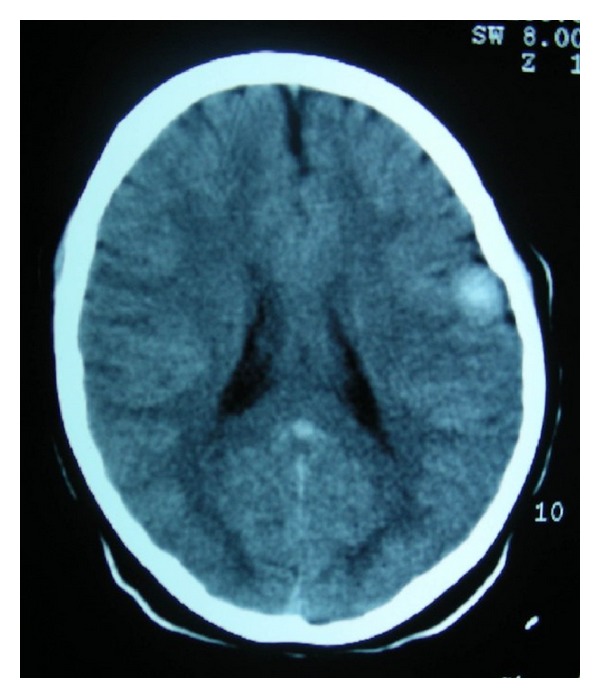
Preoperative axial CT scan showing a well-circumscribed superficial nodular hyperdense mass measuring 1.8 × 1.6 cm in the left frontoparietal lobe with remodeling of the inner table of the skull.

**Figure 2:**
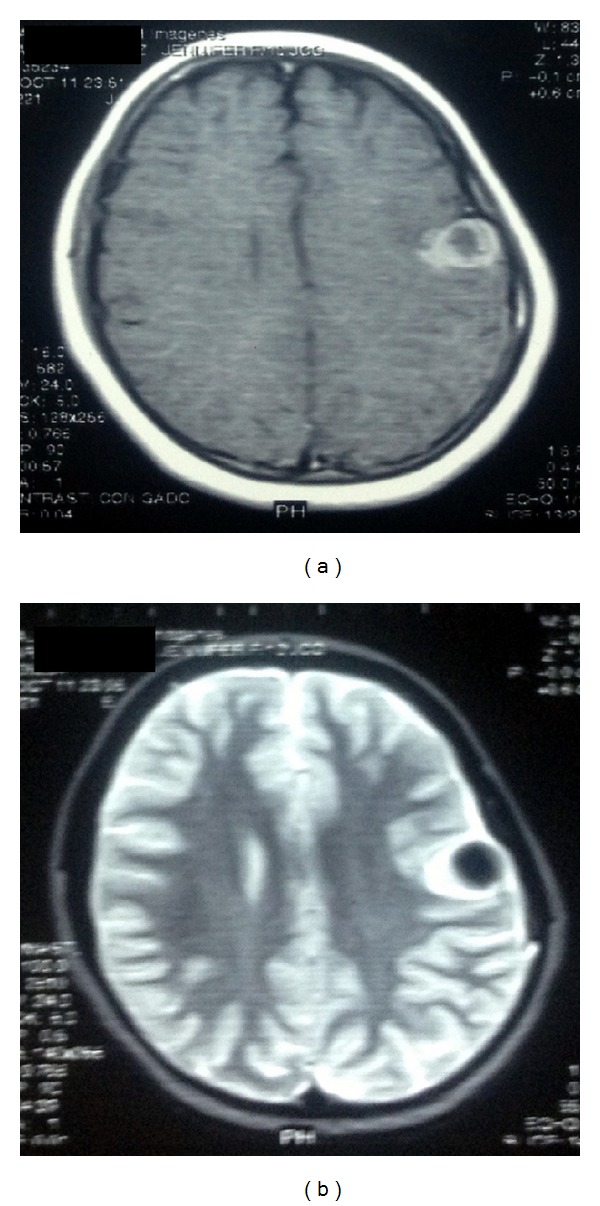
Preoperative MRI: T1-weighted axial image showing an iso-hypointense lesion (a) with peripheral contrast enhancement and a T2-weighted axial hypointense image (b) without mass effect or ventricle involvement.

Preoperative computed tomography (CT) scan showed a well-circumscribed superficial hyperdense solid nodule in the left frontoparietal lobe with remodeling of the inner table of the skull (Figure 1). The MRI showed a lesion that was isointense in T1 with perilesional contrast enhancement and hypointense in T2 (Figure 2). No mass effect or ventricle involvement was seen.

The patient underwent surgery and the mass was totally removed.

The girl received no postoperative radiation therapy or chemotherapy. She is currently being followed up and does not show any clinical evidence of relapse two years after surgery.

## 3. Macroscopic Analysis and Histology

**Figure 3:**
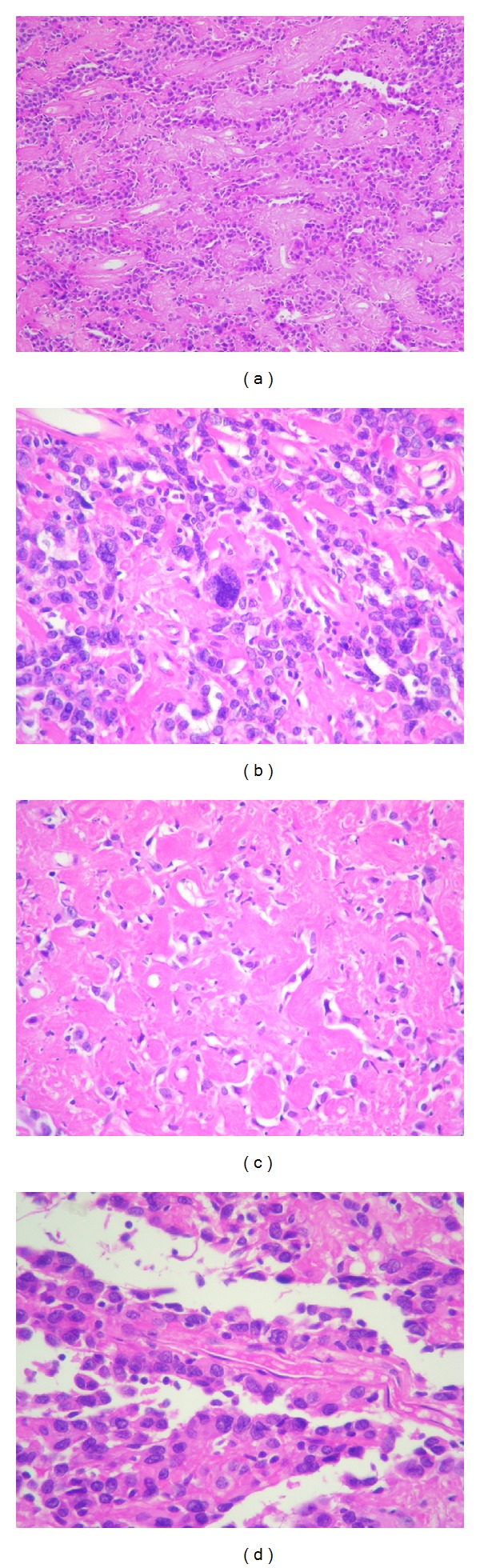
Pathological findings. The tumor is composed of glial cells arranged in a prominently hyalinized collagenous stroma (a). Some of the glial bi- or multinucleated cells show moderate pleomorphism (b). In some areas the stromal component and hypocellularity are more evident (c). A pseudopapillar pattern is observed in some areas (d). Scarce perivascular lymphocytes are visible. ((a)–(d): pathological findings: (a) 40x; (b) and (d): 400x; (c): 100x Hematoxylin & Eosin).

**Figure 4:**
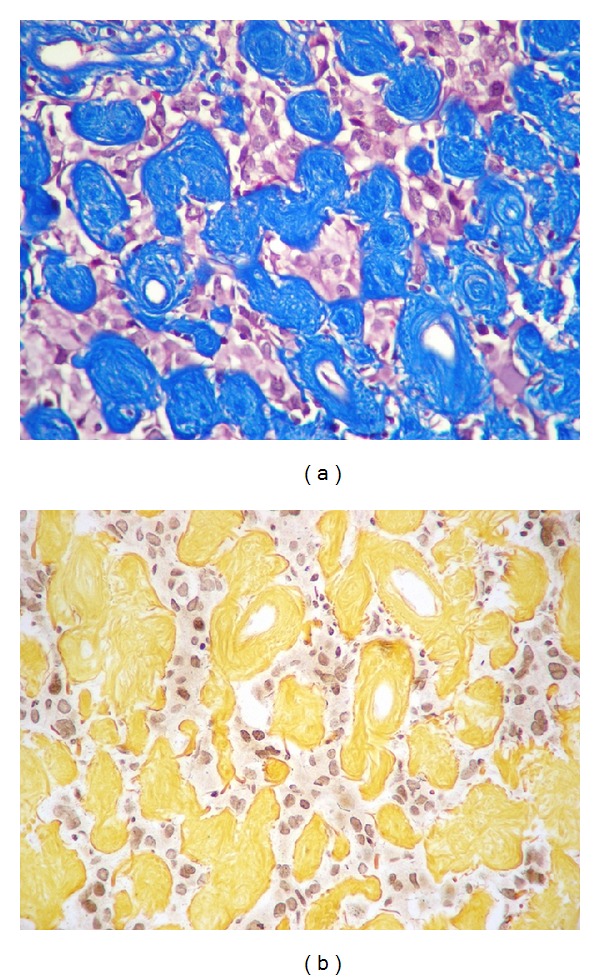
Special techniques. (a) Masson trichrome stain showing abundant acellular hyalinized connective tissue in the stroma, sometimes surrounding a vessel. Reticulum fibers are not increased (b) ((a): Masson trichrome stain; (b): silver staining: original magnification 400x).

The specimen was sent to the laboratory during surgery for intraoperative diagnosis. Macroscopically, the sample consisted of a nodule measuring 1.8 cm × 1.6 cm. The mass was white and firm. Intraoperative smear analysis showed a highly cellular tumor with moderate pleomorphism and abundant collagenous stroma. The tumoral tissue was fixed in formalin and embedded in paraffin. Five-micrometer-thick sections were cut and stained with hematoxylin-eosin (HE). As can be observed in Figure 3, microscopic examination showed a relatively well-circumscribed tumor composed of glial cells arranged in a prominently hyalinized collagenous stroma. The glial component consisted of medium- or large-sized cells, of which some were bi- or multinucleated, displaying mild to moderate pleomorphism, with eosinophilic cytoplasm and vesicular nuclei. Some cells showed elongated or irregular cytoplasmic processes. Isolated intranuclear pseudoinclusions were observed. The lesion was of variable density, showing a pseudopapillar pattern in some areas. Sparse perivascular infiltrate of lymphocytes was seen (Figure 3). Masson trichrome and silver stain for reticulin were done to stain connective tissue. Masson-trichrome-stain-positive abundant acellular hyalinized connective tissue was found in the stroma, sometimes surrounding a vessel (Figure 4).

**Figure 5:**
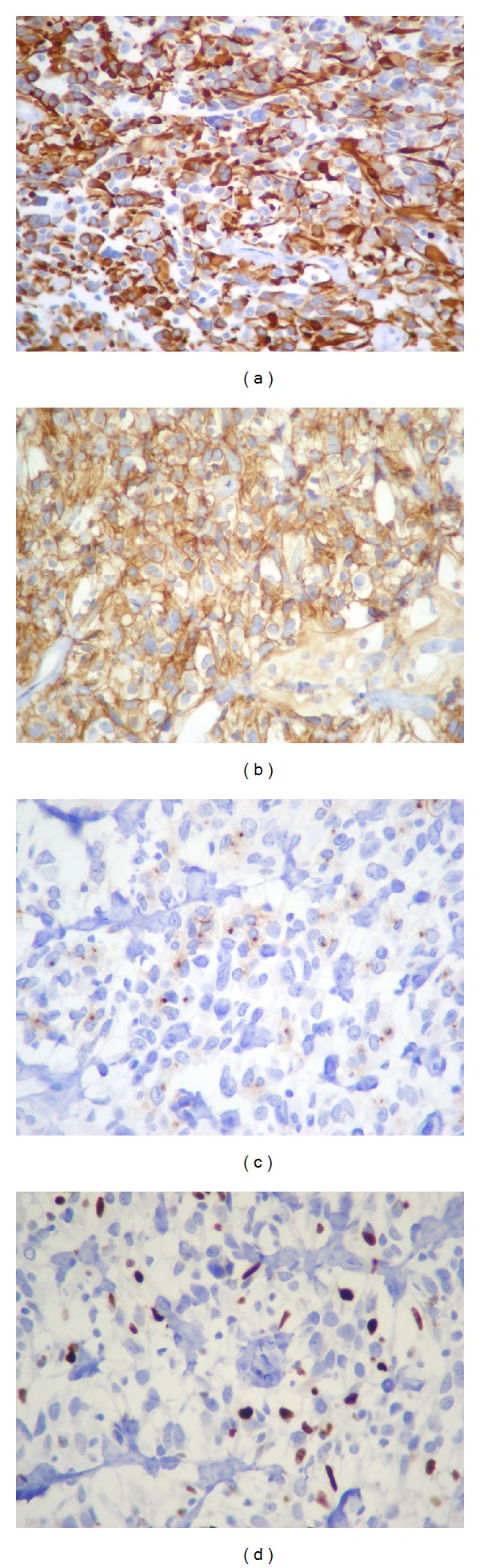
Immunohistochemical findings. Tumor cells showing diffusely positive staining with GFAP (a), CD 99 (b), vimentin, and S100. EMA staining reveals a paranuclear dot pattern in some cells (c). Proliferation index (d) is up to 10% in the cellular areas of the tumor ((a)–(d) immunohistochemistry: original magnification 400x).

Immunohistochemical staining was performed using the avidin-biotin immunoperoxidase method for glial fibrillary acidic protein (GFAP; monoclonal antibody, DAKO, Carpinteria, CA, USA; 1 : 100), S100 protein (monoclonal antibody, Novocastra, Newcastle upon Tyne, UK; 1 : 40), synaptophysin (monoclonal antibody, DAKO; 1 : 40), neurofilaments (monoclonal antibody, DAKO; 1 : 50), vimentin (monoclonal antibody, Biogenex; 1 : 100), CD99 (monoclonal antibody, clone 12E7, DAKO; 1 : 50), epithelial membrane antigen (EMA) (monoclonal antibody, clone E29, DAKO; 1 : 50), p53 (monoclonal antibody, Novocastra, Newcastle upon Tyne, UK; 1 : 50), and Ki-67 (monoclonal antibody, DAKO; 1 : 50). Immunohistochemical examination revealed positive staining for GFAP, CD 99, S100, and vimentin. EMA staining showed a paranuclear dot pattern in only few cells in isolated areas. None of the cells expressed synaptophysin, neurofilaments, or p53. Ki-67 expression was up to 10% in the cellular areas of the tumor (Figure 5).

There were no areas of necrosis, microvascular proliferation, or significant inflammatory cell infiltrates. Mitotic index was <1 per 10 high-power fields.

These histological and immunohistochemical findings, showing a glial component with a collagen-rich stroma, were consistent with a desmoplastic glioma.

## 4. Discussion

Gliofibroma was first described by Friede in a three-year-old child in 1978 [1]. Since then, only 25 cases of gliofibroma have been reported in the pediatric population. In Table 1, the cases that have been published up to now and our case are summarized.

This kind of tumor affected children more than adults, so far, and occurred both in the brain and in the spinal cord. In children, the tumors were hemispheric lesions in less than half of the cases. In 1984, Iglesias et al. reported a prenatally diagnosed congenital case [2].

Gliofibroma is not listed as a distinct entity in the current WHO classification of CNS tumors [3]. Biological behavior, histogenesis, and prognostic factors are still undetermined due to the lack of cases described.

This rare tumor is composed of a glial and a mesenchymal component, of which the latter is consistently benign. The majority of these neoplasms have a benign histology and show no recurrence or metastasis after resection. GFAP and reticulin staining clearly show the biphasic appearance of these tumors. Necrosis or microvascular proliferation is not a typical feature, and nuclear pleomorphism and increased mitotic activity are rarely seen [4]. A low Mib1 labeling index has been associated with good prognosis. In one case, an Mib1 index of up to 35.8% has been described; however, in this patient, follow-up was too short for a meaningful interpretation of this value as to prognosis [5].

Fibroblasts, astrocytes, histiocytes, myofibroblasts, endothelial and Schwann cells, or multipotent glial/mesenchymal progenitor cells have been proposed to be involved in the genesis of the mesenchymal formation of this tumor [6–8]. Only primary tumors have been reported, except in one case in which the tumor is proposed to have arisen from hamartoma-like lesions [9].

Different authors define two subgroups of gliofibroma: desmoplastic astrocytoma, in which collagen is produced by the glioma cells themselves, and mixed glioma/fibroma, in which collagen is produced by mesenchymal cells [10, 11]. Nevertheless, the differentiation between these two subgroups remains controversial.

Molecular data are still insufficient; however, genetic analysis revealed a loss of heterozygosis in chromosomes 10 and 17 [12]. Further molecular studies are currently ongoing.

Ultrastructural studies have been performed to determine the mesenchymal component. On electron microscopy, Friede's case lacked fibroblast-like cells, although basement membranes were occasionally observed between the astrocytic cells and the collagen fibers. Snipes et al. described a basement membrane clearly separating the glial from the mesenchymal component [7]. Iglesias et al. found a common basement membrane investing both the astrocytes and the fibroblasts in their case [2]. Other authors were not able to identify basement membranes between collagen and glial fibers [13].

There are reports of fibrous connective tissue that was positive for Masson's trichrome or Gomori's reticulin staining [14].

Histologically, gliofibromas should be distinguished from other collagen-producing tumors of the central nervous system, including desmoplastic infantile astrocytoma/ganglioglioma (DIA/DIG) and gliosarcoma. Similar to gliofibroma, DIA/DIG has a prominent desmoplastic component characterized mainly by reticulin-positive staining [9, 15, 16]. Nevertheless, the tumors are considered different entities in terms of morphology, immunohistochemistry, and clinical aspects. DIA/DIG is a benign cystic tumor occurring in infants consisting of three distinctive components, a desmoplastic leptomeningeal, a poorly differentiated neuroepithelial, and a cortical component [3], while gliofibroma is mainly a solid tumor with a glial and a benign mesenchymal component. On the other hand, in gliosarcoma, both the glial and the mesenchymal components are malignant [3].

Yoshida et al. reported an interesting case presenting a variant of gliofibroma and suggested the term “desmoplastic ependymoma” to refer to [17]. The description of the tumor based on the morphological findings is quite different from the other cases published. In our case, the strong eosinophilic matrix seen in some areas, at times associated with a perivascular arrangement, and the focal DOT paranuclear EMA staining are suggestive indicators of an ependymal rather than of an astrocytic origin. Therefore, we agree with Yoshida who proposed the tumor to be an ependymoma.

Complete surgical resection appears to be the treatment of choice. The extent of surgical resection, tumor grade, organ involvement, and associated clinical findings determine the need for adjuvant chemotherapy or radiation therapy [18].

Because of the rarity of this tumor, the prognosis is still not clearly known. The longest follow-up reported is 10 years [19]. Most of the published cases of gliofibroma presented a benign histology, indolent course, and showed no evidence of disease progression [20, 21]. The tumor in the patient described by Friede had a malignant clinical behavior and the child died of the disease three months after the first manifestations [1]. Other cases with a poor outcome have been reported [1, 4, 7, 8, 22]. Sharma et al. described a case of gliofibroma with anaplastic features that behaved aggressively and the patient died 6 months after diagnosis [4]. Nevertheless, the prognosis seems to be related to the location and grade of resection [6, 8].

In summary, the tumor described here is a rare entity with, as yet undefined, differential biological behavior. The cases reported under the name of gliofibroma could represent a spectrum of different entities regardless of their fibrous or desmoplastic aspect. Histogenesis may be astrocytic in some and ependymal, as we suggest in our case, in others.

We believe in the importance of reporting every case in order to adequately analyze and categorize the tumor in the next WHO classification.

## Figures and Tables

**Table 1:** Clinical data and outcomes of patients with gliofibroma in the pediatric population published in the literature and the case presented here.

Author/reference	Year	Age		Sex	Location	Surgery	Adjuvant therapy	Outcome (time of follow-up)
Friede [1]	1978	3.9 yrs		F	Brainstem	Autopsy	RT CH	Died (3 mo)

Iglesias et al. [2]	1984	11 d		M	Spinal cord	PR	None	Alive (4 yrs)
Reinhardt and Nahser [9]	1984	16 yrs		F	Hemispheric	CR	None	Alive (6 mo)

Snipes et al. [7]	1991	<2 mo		F	Posterior fossa/thalamus	PR	None	Died (16 mo)
Vázquez et al. [8]	1991	9 yrs		F	Spinal cord	PR	RT	Died (18 mo)
Vázquez et al. [8]	1991	5.6 yrs		M	Spinal cord	PR	RT	Alive (2.5 yrs)
Vázquez et al. [8]	1991	11 mo		F	Hemispheric	PR	None	Alive (2 yrs)

Schober et al. [6]	1992	18 yrs		M	Hemispheric	CR	ND	ND
Iglesias-Rozas et al. [23]	1992	1.2 yrs		F	Hemispheric	CR	None	Alive (18 mo)

Rushing et al. [15]	1993	6 mo		F	IV ventricle	CR	None	Alive (2 yrs)
Cerda-Nicolas and Kepes [10]	1993	9 yrs		M	Hemispheric	CR	ND	Alive (5.5 mo)
Cerda-Nicolas and Kepes [10]	1993	4 yrs		F	IV ventricle	Bx	ND	ND

Windisch et al. [24]	1995	5 mo		M	Spinal cord	PR	None	Alive (7 mo)
Caldemeyer et al. [21]	1995	8 yrs		M	Hemispheric	ND	CH	Alive (ND)
Caldemeyer et al. [21]	1995	6 mo		F	Cerebellum	CR	None	Alive (ND)

Prayson [25]	1996	3 mo		M	Hemispheric	PR	None	Alive (3 yrs)

Mölenkamp et al. [26]	1998	ND		ND	ND	ND	ND	ND

Matsumura et al. [12]	2002	12 yrs		F	Spinal cord	CR	ND	Alive (2.9 yrs)

Suárez et al. [18]	2004	ND		ND	ND	Bx	CH	Alive (3 yrs)
Erguvan-Önal et al. [27]	2004	16 yrs		M	Hemispheric	CR	None	Alive (14 mo)

Deb et al. [28]	2006	15 yrs		ND	Brainstem	CR	None	ND

Goyal et al. [14]	2007	8 yrs		M	Hemispheric	PR	RT CH	Alive (1 yrs)
Goyal et al. [14]	2007	15 yrs		F	III ventricle	PR/CR	RT^(∗)^ CH^(∗)^	Alive (2 yrs)

Sarkar et al. [19]	2009	3 mo		F	Lateral ventricles/III ventricle	Bx	None	Alive (10 yrs)

Altamirano et al. [22]	2011	7 yrs		F	Thalamus/mesencephalon	PR	RT CH	Died (4 mo)

Gargano et al., this report	2013		10.7 yrs	F	Hemispheric	CR	None	Alive (2 yrs)

ND: no data; yrs: years; mo: months; d: days; F: female; M: male; CR: complete resection; PR: partial resection; Bx: biopsy; RT: radiation therapy; CH: chemotherapy. ^(∗)^after second surgery.

## Abbreviations

WHO: World Health Organization
CNS: Central Nervous System
CT: Computed Tomography
MRI: Magnetic Resonance Imaging
HE: Hematoxylin-eosin
GFAP: Glial Fibrillary Acidic Protein
EMA: Epithelial Membrane Antigen
DIA/DIG: Desmoplastic Infantile Astrocytoma/Ganglioglioma
ND: No data
Yrs: Years
Mo: Months
D: Days
F: Female
M: Male
CR: Complete resection
PR: Parcial resection
Bx: Biopsy
RT: Radiation therapy
CH: Chemotherapy.
